# Salvage concurrent radio-chemotherapy for post-operative local recurrence of squamous-cell esophageal cancer

**DOI:** 10.1186/1748-717X-7-93

**Published:** 2012-06-19

**Authors:** Jian Zhang, Feng Peng, Na Li, Yongmei Liu, Yong Xu, Lin Zhou, Jin Wang, Jiang Zhu, Meijuan Huang, Youling Gong

**Affiliations:** 1 Department of Thoracic Oncology and Radiation Oncology, Cancer Center, West China Hospital, Sichuan University, Chengdu, 610041, PR China; 2 State Key Laboratory of Biotherapy, West China Hospital, Sichuan University, Chengdu, 610041, PR China; 3 Department of Oncology, Second Affiliated Hospital of Anhui Medical University, Hefei, 230601, PR China

**Keywords:** Squamous-cell esophageal cancer, Post-operative local recurrence, Salvage radio-chemotherapy, Treatment outcomes, Toxicity

## Abstract

**Purpose:**

To evaluate the treatment outcome of salvage concurrent radio-chemotherapy for patients with loco-recurrent esophageal cancer after surgery.

**Methods:**

50 patients with loco-recurrent squamous-cell cancer after curative esophagectomy were retrospectively analyzed. Patients were treated with radiotherapy (median 60 Gy) combined with chemotherapy consisting of either 5-fluorouracil (5-FU) plus cisplatin (DDP) (R-FP group) or paclitaxel plus DDP (R-TP group).

**Results:**

The median follow-up period was 16.0 months. The 1-year and 3-year survival rates were 56% and 14%, respectively. The median progression-free survival (PFS) and overall survival (OS) time was 9.8 and 13.3 months respectively. There was no statistical significance of the PFS of the two groups. The OS (median 16.3 months) in the R-TP group was superior to that in the R-FP group (median: 9.8 months) (*p* = 0.012). Among the patients who had received ≥60 Gy irradiation dose, the median PFS (10.6 months) and OS (16.3 months) were significantly superior to the PFS (8.7 months) and OS (11.3 months) among those patients did not (all *p* < 0.05). Grade 3 treatment-related gastritis were observed in 6 (27.3%) and 7 (25%) patients in the R-FP and R-TP group respectively. By univariate survival analysis, the age (<60 years), TP regimen and higher irradiation dose might improve the OS of such patients in present study.

**Conclusions:**

For those patients with post-operative loco-recurrent squamous-cell esophageal carcinoma, radiotherapy combined with either FP or TP regimen chemotherapy was an effective salvage treatment. Younger age, treatment with the TP regimen and an irradiation dose ≥60 Gy might improve the patients’ treatment outcome.

## Introduction

Curative esophagectomy with radical lymph node dissection is the primary treatment for early stage esophageal carcinoma [1,2]. However, the 5-year survival rate remains only around 40% [3]. Loco-regional recurrence still is the major type of failure in those patients following surgery [4,5]. Depending on performance status, there are a number of patients which might tolerate the salvage treatment. In these patients, a potential for cure still exists.

Since 2000, radiotherapy, chemotherapy or chemo-radiotherapy have been demonstrated as the possible salvage treatment for post-operative local recurrent esophageal carcinoma, with reported the median OS of 7.0-16.0 months [6-16]. In a phase II trial, Jingu *et al.* from Japan reported that radiotherapy (60 Gy/30 fractions) combined with nedaplatin and 5-FU is a safe and effective salvage option for loco-recurrent esophageal carcinoma, achieving an impressive median OS of 39 months [17].

In our experiences, the patients with loco-recurrent esophageal carcinoma and good performance status could be considered as the potentially curative ones. Radio-chemotherapy consisting of fluorouracil (5-FU)/DDP (FP) or paclitaxel/DDP (TP) regimen has been used as the salvage and definitive treatment in our practice, according to the National Comprehensive Cancer Network (NCCN) Guidelines [18].

In this study, we retrospectively evaluated the survival of the identical patients treated with salvage concurrent radio-chemotherapy, in order to analyze the impacts of the chemotherapy regimen (FP or TP) and the irradiation dose on the treatment outcome of post-operative local recurrences of squamous-cell esophageal carcinoma.

## Patients and methods

### Patients’ data

A loco-regional recurrence was defined as anastomotic recurrence or lymph node metastasis in supraclavicular and mediastinum regions; only in patients with an initial diagnosis of lower thoracic carcinoma, the abdominal lymph node metastasis was considered as local recurrences. Between March 2005 and December 2009, a total of 50 esophageal carcinoma patients received chemo-radiotherapy for loco-regional recurrence at West China hospital and Second Affiliated Hospital of Anhui Medical University. Each patient had undergone an R0 resection including extended lymph node dissection and had histologically proven squamous-cell eaophageal carcinoma. All of the patients gave their informed consent before treatment, which was in accordance with the Declaration of Helsinki [19] and also approved by the Ethics Committee of our hospitals.

The basic and clinical characteristics of the studied patients are summarized in Table 1. The median age of the patients was 54.2 years (range: 39-64 years); most of them were male and with the Eastern Cooperative Oncology Group (ECOG) performance status score 0-1 (47/50, 94.0%). The initial tumor stage (Staging system, American Joint Committee on Cancer) [20] after surgery in the present study were 17 stage I-II and 33 stage III-IV respectively. The median time between surgery and recurrence was 13.0 months (range: 5.0-32.0 months). Local recurrence was diagnosed by computed tomography (CT), upper gastrointestinal endoscopy and ultrasonography. There were 7 (12.7%), 23 (41.8%), 19 (34.5%) and 6 (10.9%) recurrences in anastomotic, supraclavicular, mediastinal and abdominal regions respectively, and 5 patients were confirmed having 2 recurrent sites respectively.

**Table 1:** Basic and clinical characteristics of the patients in present study (n = 50)

**Characteristics**	**Number of patients (%)**
***Age (years)***	
Median (range)	54 (39–64)
***Gender***	
Male/Female	42 (84.0)/8 (16.0)
***ECOG***^***a***^***performance status***	
0-1	47 (94.0)
2	3 (6.0)
***Pathology***	
Squamous-cell carcinoma (SCC)	50 (100.0)
***Tumorstage***^***b***^***after surgery***	
I-II	17 (34.0)
III-IV	33 (66.0)
***Time form surgery to recurrences (months)***	
Median (range)	13.0 (5.0-32.0)
***Sites of recurrence***^***c***^	
Anastomotic	7 (14.0)
Supraclavicular lymph nodes	18 (36.0)
Mediastinal lymph nodes	15 (30.0)
Abdominal lymph nodes	5 (10.0)
Supraclavicular/mediastinal lymph nodes	4 (8.0)
Supraclavicular/abdominal lymph nodes	1 (2.0)

^*a*^: Eastern Cooperative Oncology Group; ^*b*^: Staging system, 6^th^ edition, American Joint Committee on Cancer, 2002; ^*c*^: 4 patients with supraclavicular and mediastinal lymph nodes recurrence, 1 patients with mediastinal and abdominal lymph nodes recurrence.

### Salvage radio-chemotherapy

#### Radiotherapy

All patients underwent initial CT simulation, then the three-dimentional conformal radiotherapy (3D-CRT) and were usually applied for treament. Intensity-modulated radiotherapy (IMRT) was used if any supraclavicular lymph node was included as a target. The gross tumor volume (GTV) included all known gross disease as determined by the imaging and endoscopic findings. The clinical target volume (CTV) was defined as the GTV plus a 2-3 cm radial margin. If the target was coutoured in the supraclavicular region, the correlated lymphatic drainage regions was coutoured as the CTV, extending to the cricothyroid membrane. The planning target volume (PTV) was defined as the CTV plus a 0.5 cm margin in all direction, respectively. The patients received a conventional-fraction schedule: 1.8-2.0 Gy per fraction and 5 fractions per week with a 6-MV linear accelerator. As shown in Table 2, the median irradiation dose for the PTV was 60 Gy, with a range of 50.4-64 Gy. The dose constraint for the spinal cord was a maximum dose < 45 Gy. For lungs, the mean dose and V_20_ were limited within 15 Gy and 30% respectively.

**Table 2:** Response to treatment

	**Complete response (CR)**	**Partial response (PR)**	**Stable disease (SD)**
R-TP group	5 (17.9%)	15 (53.6%)	8 (28.3%)
R-FP group	5 (22.7%)	11 (50.0%)	6 (27.3 %)

#### Chemotherapy

The chemotherapy and radiotherapy started at the same day. The regimens consisting of either 5-FU 500 mg/m^2^/day for five days plus DDP 75 mg/m^2^ on day one per 4 weeks or paclitaxel 135 mg/m^2^ and DDP 75 mg/m^2^ on day one per 3 weeks. Only the grade 3 or higher treatment-related esophagitis were observed and if prolonged, the chemotherapy was discontinued; otherwise the chemotherapy was suspended until recovery and reduced the regimen dose by 25% in the subsequent cycle.

### Treatment assessment

Evaluation of treatment response was carried out according to Response Evaluation Criteria in Solid Tumors (RECIST criteria) [21]. Disappearance of the all GTVs was designated to indicate complete response (CR) on CT persisting for more than 4 weeks. A partial response (PR) was defined as a minimum of a 30% decrease in the sum of the longest diameter of target lesions. A disease was defined stable (SD) where there was neither a sufficient shrinkage to qualify for a PR nor a sufficient increase in the target lesions, and progressive (PD), when there was at least a 20% increase in the sum of the longest diameter of the target lesions or appearance of new lesions. Follow-up evaluations were performed every 2 to 3 months for the first year and every 6 months thereafter by CT.

Toxicities were evaluated according to the National Cancer Institute Common Toxicity Criteria version 3.0.

### Statistical methods

Statistical analyses were performed using the SPSS software (version 13.0). The progression-free survival (PFS) time was measured from the date the treatment began to the date of the disease progression and the overall survival (OS) time was considered from the start of treatment to date of data analysis or date of loss from follow-up for patients alive. Patients without disease relapse or progression who discontinued the study for any reason were censored at the last on study tumor assessment date. The rates of PFS and OS curves depending on the different factors were calculated using the method of Kaplan-Meier analysis and were compared using a log-rank test. A *p* value < 0.05 was considered with statistical significance. Also, Cox's proportional hazards regression model was used for univariate survival analysis. Patient age, gender, staging after surgery, time interval between surgery and recurrence, irradiation dose, chemothrapy regimen, tumor response to treatment were put into univariate analysis. Due to the small patient numbers, multivariate analysis was not performed.

## Results

The median follow-up time for the studied patients was 16.0 months (range: 10.0-44.0 months) and the median time interval between surgery and recurrence was 13.0 months (range: 5.0-32.0 months). All patients completed the radiotherapy treatment. In present study, 72.7% (16/22) and 75% (21/28) patients had received 2 cycles of chemotherapy in the FP and TP group respectively. And the remaining patients had received at least 1 cycle of chemotherapy.

### Responses to treatment

All patients were assessed as having had a response (details shown in Table 2). In the R-FP group, 5 (22.7%), 11 (50%) and 6 (27.3%) patients showed CR, PR and SD, respectively. And in the R-TP group, these numbers were 5 (17.9%), 15 (53.6%) and 8 (28.3%) respectively. The overall responses were 72.7% (16/22) and 71.7% (20/28) in the R-FP and R-TP group respectively.

### Follow-up

Patient follow-up studies continued until December 2011, with no one lost to follow-up. The 1-year and 3-year survival rates were 56% and 14% respectively. The median PFS of the whole group was 9.8 months (range: 4.7-41.0 months) and the median OS of all patients was 13.3 months (range: 5.4-44.0 months).

**Figure 1:**
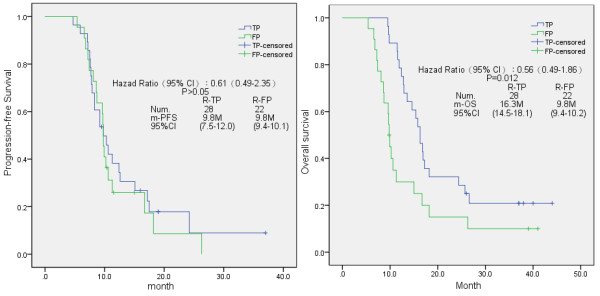
Kaplan-Meier analysis of progression-free survival (PFS) and overall survival (OS) in the present study, according to the chemotherapy regimen the patients received.

In sub-group analysis, the median PFS and OS were 9.8 months [95% confidential interval (CI) 9.4-10.1 months] and 9.8 months (95% CI 9.4-10.2 months) in the R-FP group respectively (Figure 1). The patients receiving the TP regimen had the similar outcomes of the PFS (9.8 months, 95% CI 7.5-12.0 months) but a significant improvement of the OS (16.3 months, 95% CI 14.5-18.1 months) (*p* = 0.012), compared to those patients receiving the FP regimen.

**Figure 2:**
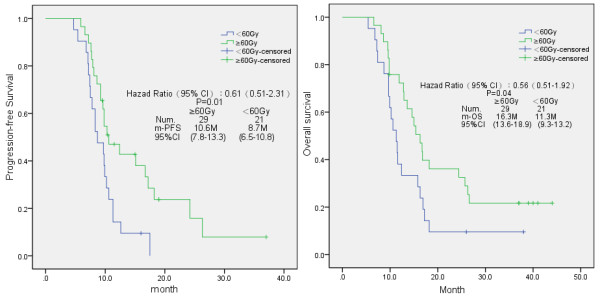
Kaplan-Meier analysis of progression-free survival (PFS) and overall survival (OS) in the present study, according to the irradiation dose the patients received.

In addition, the irradiation dose had a clear impact on the treatment outcomes in these evaluated patients (Figure 2). Patients receiving more than 60 Gy irradiation dose had significantly prolonged period in PFS (10.6 months, 95% CI 7.8-13.3 months) and OS (16.3 months, 95% CI 13.6-18.9 months) than those patients who received an irradiation dose less than 60 Gy (for PFS: 8.7 months, 95% CI 6.5-10.8 months and for OS: 11.3 months, 95% CI 9.3-13.2 months) respectively (*p* = 0.01 and 0.04, respectively).

### Treatment-related toxicities

All the patients were evaluated for tretment-related toxicities (Table 3). The combination of radiotherapy and chemotherapy (either FP or TP regimens) were proved to be tolerable. The most common toxicities were the treatment-related gastritis and neutropenia. Grade 3 treatment-related gastritis were observed in 6 patients (27.3%) and 7 patients (25.0%) in the R-FP and R-TP group, respectively. Grade 3 neutropenia were observed in 7 (31.8%) and 6 (26.4%) patients in the R-FP and R-TP group, respectively. The other major grade 2 toxicities included the neutropenia, anemia and nausea/vomiting/diarrhea. One patient receiving R-TP treatment had the grade 3 vomiting (3.6%). No grade 4 or 5 toxicity was recorded among all the patients.

**Table 3:** **Treatment-related toxicities (number = 22 and 28 in R-FP and R-TP group respectively)**^***a***^

**Toxicities**			
	**Grade 1**	**Grade 2**	**Grade 3**
Neutropenia	3 (13.6)/5 (17.9)	12 (54.5)/17 (60.7)	7 (31.8)/6 (21.4)
Anemia	8 (36.4)/10 (35.7)	14 (63.6)/18 (64.3)	0/0
Thrombocytopenia	17 (77.3)/18 (64.3)	5 (22.7)/10 (35.7)	0/0
Digestive tract side-effects^*b*^	3 (13.6)/7 (25)	19 (86.4)/20 (71.4)	0/1 (3.6)
Treatment-related esophagitis	2 (9.1)/3 (10.7)	14 (63.6)/18 (64.3)	6 (27.3)/7 (25.0)

^*a*^: Data presented as number in the R-FP group (%)/number in the R-TP group (%); ^*b*^: Including nausea, vomiting and diarrhea.

### Univariate survival analysis

Due to the small number of the evaluated, only the univariate analysis was performed according to the basic and clinical characteristics of the patients. The details were shown in Table 4. The gender, disease stage after surgery, the responses to the treatment and irradiation dose did not significantly affected the survival time. While the patients’ age (<60 years), the chemotherapy regimen (TP) and irradiation dose more than 60 Gy showed the trends which could improve the overall survival of the patients in present study respectively (*p* = 0.048, 0.025 and 0.041, respectively).

**Table 4:** **Prognostic factors by log-rank test and univariate survival analysis**^***a***^**in present study**

**Factors**	**Group**	**Number**	**Median OS**^***b***^ **(months)**	**Log-rank test**	**Univariate analysis**
				***p*value**	***p*value**
**Age**	< 60 years	16	14.3	0.042	0.048
	≧60 years	34	11.9		
**Gender**	Male	42	15.0	0.365	0.334
	Female	8	10.6		
**Staging after surgery**	I/II	17	17.2	0.355	0.359
	III/IV	33	13.8		
**Chemotherapy regimen**	TP	28	16.3	0.012	0.025
	FP	22	9.8		
**Response to treatment**	CR/PR	40	15.0	0.071	0.072
	SD	10	12.8		
**Irradiation dose**	≧60 Gy	29	16.3	0.040	0.041
	< 60 Gy	21	11.5		

^***a***^: Cox's proportional hazards regression model; ^*b*^: Overall survival.

## Discussion

Loco-regional recurrences after intial surgery in patients with esophageal cancer remain a serious challenge to clinical oncologists. The NCCN Guidelines pointed out that a highly selected group of patients with local-regional tumor recurrence after initial surgery may be considered fit and able to tolerate concurrent radio-chemotherapy with a potential for cure [18]. In a line with the previous studies, our data indicated that salvage concurrent radio-chemotherapy was an active and promising treatment strategy for such patients, reaching a median OS of 13.3 months with tolerable side-effects.

The present protocol of concurrent radio-chemotherapy was completed in 74% (37/50) of the patients, and no serious treatment related toxicities were observed. The tumor response rate was nearly 72% in R-TP and R-FP group respectively, with a 3-year survival rate of 14%. These results are very similar to those reported in previous studies [13,15]. Yamashita *et al.*[13] reported the results of radiotherapy with or without chemotherapy at an average total dose of 56.6 Gy. The median survival time was 13.8 months and 1-year survival rate was 56% in patients with patients with loco-regional recurrence of esophageal cancer after curative surgery. Recently, Baxi *et al.* from Princess Alexandra Hospital reported their treatment outcomes using DDP and 5-FU plus radiotherapy (range: 45–60 Gy) for recurrent esophageal cancer [15]. The 2-year survival rate of all patients was 21% and the median OS was 16 months. While the very impressive results of salvage radio-chemotherapy for such patients were reported by Jingu *et al.*[17] and the 3-year survival rate was 56.3% using radiotherapy combined with nedaplatin and 5-FU. But it was a small phase II study (patients’ number = 30), and these data had not been confirmed by further clinical investigations.

By sub-group analysis in the present study, the median OS of the 28 patients received R-TP regimen (16.3 months) was significantly superior than that of other patients who received R-FP regimen (9.8 months) (*p* < 0.05). According to our knowledge, there was no direct comparison between FP and TP regimen when combined with radiotehrapy in the definitive treatment for esophageal cancer. Recently, Ajani *et al.* reported that the regimen including 5-FU/DDP/paclitaxel with 50.4 Gy of radiation was associated with high morbidity, although it reached a 1-year survival rate of 75.7% in RTOG 0113 trial [22]. So far, FP regimen still was the first choice in definitive radio-chemotherapy for esophageal cancer in the NCCN guidelines [18]. The possible reason that the poorer treatment outcomes using R-FP regimen in present study was the relative lower dosage of 5-FU (500 mg/m^2^/D, D1-5 per 4 weeks). In the trial INT 0123, Minsky *et al.* established the standard dosage of 5-FU (1000 mg/m^2^/D, D1-4 per 4 weeks) in the definitive radio-chemotherapy for esophageal cancer [23]. However, considering the relative smaller body size of the Chinese population, the dosage of 5-FU was consequently decreased, similar in methodology with other Asian studies [12,17].

In addition, our data indicates that an irradiation dose of more than 60 Gy could improve not only the PFS but the OS among those patients with recurrent esophageal cancer after surgery (both *p* < 0.05). In our opinion, 50.4 Gy was the standard irradiation dose for esophageal cancer reported by Minsky *et al.*[23] in trial INT 0123 and the higher radiation dose (64.8 Gy) did not increase the survival or local/regional control. But the authors also pointed out that 63.6% (7/11) the treatment-related deaths in the high-dose arm occurred in patients who received 50.4 Gy or less, and the higher dose of irradiation might not be responsible for the increased mortality. In 2001, reports from Nemoto *et al.*[8] indicated that no significance between the patients’ survival received more than or less than 60 Gy. But the 1-year and 3-year survival rates were 45% and 20% in the higher dose arm respectively, which were superior than those of the lower dose arm (15% and 7% respectively). In the study by Baxi *et al.*[15] most patients (79%, 11/14) received irradiation dose of 58–60 Gy and the median OS (16 months) of all patients was encouraging. And in study by Jingu *et al.*[17], the irradiation dose was 60 Gy/30 fractions and the treatment outcomes (median OS of 39 months) were extremlly exciting. To data, one study has indicated that the survival was similar among the patients received an irradiation dose more or less than 60 Gy [14]. After a careful review of the literatures, the suitable irradiation dose for recurrent esophageal cancer remains unclear and requires further investigations.

With regard to the side-effects of concurrent radio-chemotherapy for post-operative esophageal cancer, the irradiation dose for the esophago-gastric anastomosis and the gastric tube should be of particularly concerm. In the present study, grade 3 treatment-related gastritis were recorded in 6 patients (27.3%) and 7 patients (25.0%) in the R-FP and R-TP group, respectively. One patient received R-TP treatment was observed having the grade 3 vomiting. As far as we know, the prediction probability of the normal tissue complication at 5% within 5 years after radiotherapy (TD 5/5) of the stomach is 60 Gy [24]. Furthermore, Nemato *et al.*[8] reported that one patient died of necrosis of the stomach 6 months after comletion of radiation therapy (66 Gy). Therefore, we avoided prescribing a dose of more than 60 Gy to the recurrence of the anastomotic sites in present study, and only recurrences of the regional lymph nodes received the irradiation dose more than 60 Gy. As a result, no serious treatment-related side-effects (gastric fistula or necrosis) were observed in the follow-up. Other common toxicities included hematological side-effects (neutropenia and anemia) and digestive tract toxicities in present study. Compared to the previous studies of patients with the locally-advanced esophageal cancer [22,23,25-27], the treatment-related toxicities of the concurrent radio-chemotherapy in present study were similar to or less than the studies mentioned above [8,12-15,17].

One issue should be mentioned here. According to the NCCN guidelines [18], also as summarized by two meta-analysis [28,29], the pre-operative chemoradiation followed by surgery is the most common strategy for patients with resectable esophageal cancer in Western countries, which could significantly improve the 3-year OS and reduce the loco-reginal recurrences. The irradiation dose in such approach is nearly 40-45 Gy. Thus, the patient who received the pre-operative chemoradiation could not be treated with such higher irradiation dose similarly to the present study (around 60 Gy) for loco-regional recurrence. The optimal treatment strategies for this specific group of patients need further clinical studies.

In conclusion, combination of radiotherapy with concurrent FP or TP chemotherapy is a safe and promising salvage treatment for loco-regional recurrence of esophageal cancer after surgery. The use of TP regimen and an irradiation dose of more than 60 Gy may improve the overall survival of these patients. However, the optimal treatment strategy (irradiation dose and chemotherapy regimen) for loco-regional recurrent esophageal cancer warrents further studies.

## Misc

Jian Zhang, Feng Peng and Na Li contributed equally to this paper.

## Competing interests

The authors declare that they have no competing interests.

## Authors’ contributions

JZ, FP and NL contributed equally in collection and analysis of data and drafting the manuscript; YL, YX, LZ, JW, JZ and MH provided the critical revision of the manuscript and the administrative support; YG provided the conception of this study and the final approval of the version to be published. And all authors read and approved the final manuscript.
